# Recent advances in predicting responses to antidepressant treatment

**DOI:** 10.12688/f1000research.10300.1

**Published:** 2017-05-03

**Authors:** Thomas Frodl

**Affiliations:** 1 Department of Psychiatry and Psychotherapy, Otto-von-Guericke University of Magdeburg, Magdeburg, Germany

**Keywords:** major depressive disorder, depression, neuroimaging, EEG, antidepressant treatment

## Abstract

Major depressive disorder is one of the leading causes of disability in the world since depression is highly frequent and causes a strong burden. In order to reduce the duration of depressive episodes, clinicians would need to choose the most effective therapy for each individual right away. A prerequisite for this would be to have biomarkers at hand that would predict which individual would benefit from which kind of therapy (for example, pharmacotherapy or psychotherapy) or even from which kind of antidepressant class. In the past, neuroimaging, electroencephalogram, genetic, proteomic, and inflammation markers have been under investigation for their utility to predict targeted therapies. The present overview demonstrates recent advances in all of these different methodological areas and concludes that these approaches are promising but also that the aim to have such a marker available has not yet been reached. For example, the integration of markers from different systems needs to be achieved. With ongoing advances in the accuracy of sensing techniques and improvement of modelling approaches, this challenge might be achievable.

## Introduction

Major depressive disorder (MDD) is a frequent and disabling disorder with prevalence rates of about 16% ^1^. Although successful psychotherapy and pharmacotherapy are available, it usually takes at least 2 to 3 weeks until symptoms improve. The first antidepressant may be beneficial in only 50 to 60% of cases. In the Sequenced Treatment Alternatives to Relieve Depression (STAR*D) study, more than 40% of patients with MDD did not achieve remission even after two optimally delivered trials of antidepressant medications ^2^. Thus, biomarkers would be warranted that predict response to a specific class of antidepressants or a therapy method (psychotherapy, pharmacotherapy) and could help stratifying patients for tailored therapies and, in turn, would shorten the time of being depressed. Possible neuroimaging, electrophysiological, genetic, and proteomic markers will be reported, and the main focus will be on recent developments. Other forms of therapies, including transcranial magnetic stimulation, electroconvulsive therapy (ECT), deep brain stimulation, or therapy with ketamine, also exist but will not be reviewed here.

## Functional neuroimaging

Interestingly, brain activation and particularly neural correlates of emotional processing could have predictive value for determining patients who will respond to treatment against depression. A detailed overview on this topic can be seen in Frodl ^3^. In studies with positron emission tomography (PET), it was found that the subgenual anterior cingulate cortex (sgACC) may serve as a biomarker for treatment response. Metabolism in the sgACC predicted response to antidepressant therapy ^4, 5^. Later research demonstrated that this seems not to be restricted to the sgACC. Decreased metabolism in the insular cortex also predicted response to treatment in patients with MDD ^6^. Response to cognitive behavioral therapy (CBT) has been found to be associated with metabolic increases in the hippocampus and posterior cingulate cortex (pCC) and decreases in dorsal, ventral, and medial frontal cortices ^7^. Response to selective serotonin reuptake inhibitors (SSRIs) was related to decreases in glucose metabolism in ventral regions of the prefrontal cortex (PFC) ^8, 9^ and increases in the temporal cortex ^10, 11^. Before initiating treatment, responders to pharmacological treatment showed greater activation in the dorsomedial PFC (dmPFC), pCC, and superior frontal gyrus when viewing negative emotional pictures as compared with being in resting condition. Moreover, response to therapy was predicted by activations in the caudate nucleus and insula contrasted for emotional compared with neutral stimuli ^12^. In a meta-analysis, responders and non-responders to paroxetine and a combination of antidepressants differed in activation of the hippocampus-orbitofrontal cortex (OFC)-anterior cingulate cortex (ACC)-lateral PFC network ^13^. Recently, network analyses were more used in clinical functional magnetic resonance imaging (fMRI) research since functional connectivity and alterations in network characteristics might offer signals closer to biological brain functions than the contrast of blood oxygen level-dependent (BOLD) response between different conditions in a selected brain region. Interestingly, good OFC connectivity observed before treatment was shown to be associated with response to antidepressants ^14^. In a trial with mirtazapine and venlafaxine, distinct therapy effects on BOLD response and differential response prediction with baseline BOLD response were detected. Patients who received venlafaxine significantly decreased their BOLD responses in the hippocampus, basal ganglia, thalamus, and cerebellum, whereas patients who received mirtazapine significantly increased their BOLD responses in the middle cingulate gyrus and supplementary motor area. Better response to venlafaxine was associated with larger BOLD responses in the left fusiform gyrus at baseline, and a better response to mirtazapine was associated with smaller BOLD responses in the right rolandic operculum at baseline ^15^.

A few studies exist that investigated the use of fMRI to predict treatment response to psychotherapy. Amygdala hyperactivation and ACC hypoactivation during fMRI predicted response to CBT ^16, 17^.

In one of the latest fMRI trials, 80 patients with MDD and 34 healthy controls were included ^18^. All MDD participants either were antidepressant medication-naïve or, if previously prescribed an antidepressant medication, had undergone a washout period of at least 1 week (five half-lives). Patients were randomly assigned to receive the antidepressants sertraline, citalopram, or venlafaxine. The first two are SSRIs, inhibiting reuptake of serotonin, and the last one is a serotonin-norepinephrine reuptake inhibitor (SNRI). In fMRI using a subliminal emotional face paradigm, responders were characterized by lower amygdala responses due to subliminal positive or threat-related emotional face stimuli. During subliminal assessment of sad faces, a differential effect was seen with regard to the medication group used. Here, pre-treatment amygdala activation to sad facial expressions was specifically and differentially predictive of which participants failed to respond to a specific antidepressant, the SNRI venlafaxine-XR. The overall accuracy to classify responders and non-responders correctly was 75%. Thus, using fMRI could lead to a better prognostic value than current practice based on clinical impressions ^18^.

Therefore, patients responding to antidepressant therapy seem to differ from patients not responding to antidepressants with regard to their fMRI BOLD responses. Depending on the task used and region investigated, response could be predicted by either hyper- or hypo-activation. Thus, responders might be likely to be identified by fMRI. However, this link and the underlying neurochemical changes of functional brain changes require further exploration. Taken together, these findings are promising and emphasize that functional neuroimaging could be developed further to predict treatment response.

It has to be mentioned that other magnetic resonance imaging (MRI) techniques might also have potential to predict therapy response. For example, smaller hippocampal volumes in patients with depression predicted a worse illness course compared with patients with larger hippocampal volumes ^19^. A recent study confirmed that hippocampal volumes and also sgACC volumes can predict treatment response to ECT in patients with major depression ^20^.

## Electrophysiology

Event-related potentials (ERPs) and electroencephalogram (EEG) recordings have been studied for their ability to predict treatment response, and some measures have been demonstrated to be promising. The ERP is a waveform in brain activity that is related to an event (for instance, an auditory or visual stimulus). Most research has been focused on the ERPs P300 (P3) and N100 (N1); for a review, see Arns and Olbrich ^21^. A very recent study using a multi-center design included 1,008 patients with MDD. Three different treatment options (venlafaxine, sertraline, and citalopram) were compared. Male responders to venlafaxine-XR showed significantly more negative N1 amplitudes measured before treatment initiation than non-responders. The effect sizes were large (for example, with d Fz = 0.89). Furthermore, N1 amplitudes at Fz correlated significantly with percentage improvement of depression ^22^.

A lot of effort has been undertaken to establish the loudness dependence of auditory evoked potentials (LDAEP) N1 and P2 as a marker for the central serotonergic system and thus as a marker for therapy response to SSRIs ^23^. Recently, 51 patients with depression were investigated by using LDAEP and it was confirmed that patients with steep LDAEP N1/P2 responded better to antidepressant therapy ^24^. Response to SSRIs was also found to be associated with a higher slope of LDAEP in patients with MDD ^25^.

In line with the predictive ability of sgACC PET activity, sgACC EEG theta activity was also found to be associated with therapy response. This is understandable since rostral anterior cingulate cortex (rACC) theta current density has been shown to correlate with rACC metabolism ^26^. Patients showing higher pre-treatment theta activity responded better to antidepressants ^27– 30^.

EEG power analyses might have further abilities to guide differential treatment. For example, relatively greater right frontal alpha in women was associated with a better response to the SSRIs escitalopram and sertraline in the iSPOT-D (International Study to Predict Optimized Treatment–Depression) trial. No such effect was found for venlafaxine-XR ^31^.

Another EEG measure with potential for therapy response is arousal system activity. In the study cohort by van Dinteren *et al*. ^22^, MDD patients with response or remission after SSRI treatment showed significant differences in central nervous system arousal slope over time as compared with non-responders or non-remitters at baseline. A faster decline of central nervous system arousal predicted a positive outcome following SSRI treatment but this was not the case in the SNRI group. In patients who received SNRIs, a significantly larger increase of autonomic nervous system arousal predicted response ^32^.

The advantage of EEG/ERP is its good availability to a much wider population and relatively cheap costs in comparison with neuroimaging and thus this technique should be used more to develop treatment response prediction tools. Also, it has to be noted that dynamic aspects of early changes during treatment initiation might provide better individual biomarkers than an assessment carried out once before starting treatment.

Combining different methods might increase the likelihood to be able to predict therapy response. In a previous study, patients with major depression (n = 20) were investigated by using both resting EEG and LDAEP before treatment with either citalopram or reboxetine. Differences between responders and non-responders were found in the rACC in the theta-frequency range. Higher LDAEP values were detected in responders versus non-responders to citalopram ^33^.

## Genetics and gene expression

Since the serotonin transporter is one of the most important targets for antidepressants (at least for SSRIs), it was a primary step to study whether genetic polymorphisms in the serotonin transporter gene have any predictive power for response to SSRIs. Interestingly, it could be demonstrated that the polymorphism in the promotor region of the serotonin transporter gene (5/HTTLPR) was associated with response to fluvoxamine ^34^ and paroxetine ^35^.

For over a decade, studies investigated the predictive value of single polymorphisms for antidepressant response and a breakthrough was not happening. Thus, a next step was to look at interactive effects of such single-nucleotide polymorphisms (SNPs). In a recent study using a detection sample of 239 cases with MDD completing a trial with SSRIs, a predictive model comprised of haplotypes and polymorphisms related to serotonin synthesis, serotonin transport, glutamate receptors, and GABA synthesis was created ^36^. This model was tested in a confirmation sample of 176 SSRI-treated patients and in a cross-over sample of non-SSRI-treated patients. Predictive values were 85% for responders and 86% for non-responders, compared with prior probabilities of 66% for observed response and 34% for observed non-response in those cases. So SSRI response was associated with polymorphisms in serotonin, glutamate, and GABA-related genes ^36^.

Moreover, in an analysis of 225 patients from a study of the European Group for the Study of Resistant Depression, 12 SNPs and clinical variables were integrated. A model using 3 SNPs and a clinical variable was seen to be most successful in predicting therapy response. In this study, patients received different agents since the inclusion definition was that they should have had at least one antidepressant treatment with sufficient duration and dose and thus no specific trial had been carried out. The study demonstrates that combining different genetic variables and clinical data and analyzing them with novel statistical tools for interaction analysis can increase the predictive power for therapy response ^37^.

Lopez *et al*. showed that baseline expression of microRNA (miRNA) miR-1202 was lower in patients with depression who subsequently responded to an 8-week regimen of the SSRI citalopram ^38^. This study opened a promising route for research to treatment response using miRNA and other RNA expression measures.

Peripheral gene expression might be a useful tool to predict therapy response and non-response. From an open-label uncontrolled 12-week trial with escitalopram, 87 patients with MDD were included in the gene expression analysis since they finished the whole study period and had good-quality RNA samples at the baseline, 4-week, and 12-week time points. Some interesting transcripts were found to be significantly upregulated in responders after 4 weeks and after 12 weeks ^39^.

In a discovery sample of 34 patients with MDD treated with citalopram, two replication cohorts with similar sample and study characteristics were used. Via co-expression analysis, it was found that miR-135 and miR-16, two miRNAs potentially associated with stress resiliency and antidepressant response ^40^, were associated with clinical improvement ^41^.

In a further study investigating miRNAs, it was found that two miRNAs (let7b and let7c) were lower in patients with depression ^42^. In particular, those miRNAs are regulators of the PiR3-AKT pathway upstream from mechanistic target of rapamycin (mTOR) that previously was found to be associated with response to ketamine treatment. Since these miRNAs were particularly low in ECT non-responders, they might serve as biomarkers for treatment resistance to ECT. However, this needs to be confirmed in larger samples ^42^.

Limitations of the genetic work are that findings have rarely been replicated. Some studies have large sample sizes but have shown only weak predictors of response and it is hard to replicate these findings since, first, it is time-consuming to obtain such large samples and, second, it is difficult to confirm the same SNPs or RNA expression signatures again in a new study, when they did show only weak signals in the previous study. Some of the studies showing either genomic or gene expression predictors are much smaller or have not been replicated or both.

## Other blood measures

Easy availability to the population could be made through measures available in chemical labs. According to Thase ^43^, “Although it is now widely recognized that depression is a pro-inflammatory state and a number of contemporary studies have examined immune status in relation to antidepressant response, no consistent pattern of immune dysfunction as a biomarker of antidepressant response has been observed to date”. Uher *et al*. investigated whether C-reactive protein (CRP) did have any predictive power for antidepressant response to citalopram or nortriptyline ^44^. Patients with low pre-treatment CRP levels were significantly more responsive to escitalopram, whereas those with high CRP levels were significantly more responsive to nortriptyline ^44^. In a sample of 76 depressed patients, it was detected that baseline brain-derived neurotrophic factor (BDNF) levels significantly predicted clinical improvement in a naturalistic clinical trial ^45^. In another study, it was detected that early changes in BDNF levels predicted therapy response. According to receiver operating characteristic (ROC) analysis, the best cutoff value for the prediction of response was found to be an increase of 338 pg/mL or 126%, respectively, of plasma BDNF between baseline and day 7 ^46^.

## Conclusions

Improved MRI techniques and fMRI tasks that make possible the study of altered brain function in depression will likely be able to provide useful information on therapy effects and, in the future, may be able to predict therapy response. Also promising are EEG and ERP techniques since they are easy to access. Blood markers such as miRNAs and the integration of different genetic polymorphisms would be the most practicable biomarkers and seem promising too. However, more longitudinal research during trials and during the disease course is needed to achieve these aims.

## Abbreviations

ACC, anterior cingulate cortex; BDNF, brain-derived neurotrophic factor; BOLD, blood oxygen level-dependent; CBT, cognitive behavioral therapy; CRP, C-reactive protein; ECT, electroconvulsive therapy; EEG, electroencephalogram; ERP, event-related potential; fMRI, functional magnetic resonance imaging; LDAEP, loudness dependence of auditory evoked potentials; MDD, major depressive disorder; miRNA, microRNA; MRI, magnetic resonance imaging; OFC, orbitofrontal cortex; pCC, posterior cingulate cortex; PET, positron emission tomography; PFC, prefrontal cortex; rACC, rostral anterior cingulate cortex; sgACC, subgenual anterior cingulate cortex; SNP, single-nucleotide polymorphism; SNRI, serotonin-norepinephrine reuptake inhibitor; SSRI, selective serotonin reuptake inhibitor.
